# Supplementary material for: A Phytophthora effector recruits a host cytoplasmic transacetylase into nuclear speckles to enhance plant susceptibility
Source: eLife. 2018 Oct 22;7:e40039. doi: 10.7554/eLife.40039 (PMC6249003; doi:10.7554/eLife.40039)
Supplement: Supplementary file 1. [file elife-40039-supp1.docx]

| **primer name**  **Table supplement 2. Primers used in this study.** | **Primer 5'-3'** |
| --- | --- |
| pBINGFP2-PsAvh52-SmaI-F | CCCGGGATGCTCACGTTGACCAAGGATT |
| pBINGFP2-PsAvh52-SmaI-R | CCCGGGTCAGTTGGCCGCCTTATAAACC |
| pICH86988-FlagPsAvh52-RFP-F | TACAATTATCGATACAATGGATTACAAGGATG  ACGACGATAAGCTCACGTTGACCAAGGATTCC |
| pICH86988-FlagPsAvh52-RFP-R | GGAGGAGGCCATCCCGGGGTTGGCCGCCTT  ATAAACCTC |
| pYF2.3G-PsAvh52-sgRNA-F | CTAGCTAGCCGTAATCTGATGAGTCCGTGAGGAC |
| pYF2.3G-PsAvh52-sgRNA-R | CTAGGGTCTCGAAACCTGTTCGTCTTGCTCG  TAATGACGAGCTTACTCGTTTCG |
| pBluescriptII-PsAvh52-Up-F | ACTAGTGGATCCCCCAGAACGGGTCTGTATGTGTC |
| pBluescript II-PsAvh52-Up-R | AAGGTACGCGATCAGTTGTTACTCGGCGTT  CGTTCGATGA |
| pBluescript II-PsAvh52-down-F | TCATCGAACGAACGCCGAGTAACAACTGATC  GCGTACCTTAAC |
| pBluescript II-PsAvh52-down-R | GAATTCCTGCAGCCCGTGAGAAATGGT  GTAGCACGAT |
| pBINGFP2-GmTAP1-F | TACAAGGGTACCCCCATGTCAATGCTATCTCTTCTTCG |
| pBINGFP2-GmTAP1-R | GGATCCGTCGACCCCGTGGTTCGGGTACCAAAAC |
| pBIN-GmTAP1-mRFP-F | GCCATGGGTACCCCCGGGATGTCAATGCTATCTCTTCT |
| pBIN-GmTAP1-mRFP-R | CTCGGAGGAGGCCATCCCGGGGTGGTTCGGGT  ACCAAAACATGCC |
| pBINGFP2-GmTAP2-F | TACAAGGGTACCCCCATGCTAACCTTCAATCTCAATGC |
| pBINGFP2-GmTAP2-R | GGATCCGTCGACCCCTTAGTGATTCGGGTACCAAAACA |
| pBINGFP2-GmTAP1-NLS-F | TACAAGGGTACCCCCATGTCAATGCTATCTCTTCTTCG |
| pBINGFP2-GmTAP1-NLS-R | GGATCCGTCGACCCCCTATCCTCCAACCTTTCTC  TTCTTCTTAGGCTGGTGGTTCGGGTACCAAAAC |
| pBINGFP2-GmTAP1-NES-F | TACAAGGGTACCCCCGGGATGTCAATGCTATCTCTTCTTC |
| pBINGFP2-GmTAP1-NES-R | GGATCCGTCGACCCCCTTGTTAATATCAAGTCCAGCCA  ACTTAAGAGCAAGCTCGTTGTGGTTCGGGTACCAAAAC |
| pBINGFP2-GmTAP1^1-140^-F | TACAAGGGTACCCCCATGTCAATGCTATCTCTTCTTCG |
| pBINGFP2-GmTAP1^1-140^-R | GGATCCGTCGACCCCGGGTGACCCATGTGACTTTCTTATA |
| pBINGFP2-GmTAP1^atd^-F | TACAAGGGTACCCCCGGGATGGAGGGGAATGAACAGAA |
| pBINGFP2-GmTAP1^atd^-R | GGATCCGTCGACCCCGTGGTTCGGGTACCAAAAC |
| pGEX4T-2-GmTAP1-F | TCCCCAGGAATTCCCATGTCAATGCTATCTCTTCTTCG |
| pGEX4T-2-GmTAP1-R | CGCTCGAGTCGACCCTCAGTGGTTCGGGTACCAAAAC |
| pGEX4T-2-GmTAP2-F | TCCCCAGGAATTCCCATGCTAACCTTCAATCTCAATGC |
| pGEX4T-2-GmTAP2-R | CGCTCGAGTCGACCCTTAGTGATTCGGGTACCAAAACA |
| pET32a-PsAvh52-F | AAGGCCATGGCTGATATGCTCACGTTGACCAAGGATT |
| pET32a-PsAvh52-R | GAATTCGGATCCGATTCAGTTGGCCGCCTTATAAACC |
| pFGC5941-3UTR^GmTAP1^-AscIF | ttacaattaccatggggcgcgccTTGCAACCCTACCAAAGCTTTT |
| pFGC5941-3UTR^GmTAP1^-AscIR | ttaaatcatcgattgggcgcgccCCACAGTCAGTAAGTGGTGG |
| pFGC5941-3UTR^GmTAP1^-BamHIR | aatttgcaggtatttggatccCCACAGTCAGTAAGTGGTGG |
| pFGC5941-3UTR^GmTAP1^-BamHIF | ctctagactcacctaggatccTTGCAACCCTACCAAAGCTTTT |

| qPCR-PsAvh52-F | GATTGCCAACGGAGATTC |
| --- | --- |
| qPCR-PsAvh52-R | CACTTTCTTCCGTTTAGCC |
| qPCR-PsACTIN-F | ACTGCACCTTCCAGACCATC |
| qPCR-PsACTIN-R | CCACCACCTTGATCTTCATG |
| qPCR-3UTR^GmTAP1^-F | GGCTTTGATTAGTAGGCCTGC |
| qPCR-3UTR^GmTAP1^-R | GCCCGAAGAAAACCACTAACC |
| qPCR-GmTAP2-F | ATGCTAACCTTCAATCTCAATG |
| qPCR-GmTAP2-R | AGGAGTAACTAAGATTGGAAGG |
| qPCR-GmCYP2-F | CGGGACCAGTGTGCTTCTTCA |
| qPCR-GmCYP2-R | CCCCTCCACTACAAAGGCTCG |
| qPCR-MtN3-F | AAAAAAGAAAATAACCTAATTAAACG |
| qPCR-MtN3-R | CACCTTTACATTAATCAAACGAA |
| qPCR-PG-F | TGTGCATTATGTAGTAGAAGCCA |
| qPCR-PG-R | ACGCATAACTAGCCAGGCAT |
| qPCR-LOX-1-F | GCCCGTGTATTCATATTTTGCA |
| qPCR-LOX-1-R | CGGAAATAGGACTGCAGAATGT |
| qPCR-LOX-2-F | TGGGTGATGTTCAGAATGCT |
| qPCR-LOX-2-R | TGGTCGAATCTTGACACCAA |
| ChipPCR-MtN3-F | TATTAGGGTCAGGCACCATCGT |
| ChipPCR-MtN3-R | AGATGAATTAAGAAAGAGGTTAATTAA |
| ChipPCR-PG-F | AAAATAACATGCACAACAACATGA |
| ChipPCR-PG-R | GCAGTGAGAGTGAAGAGAAACTGT |
| ChipPCR-Lox-1-F | CTTCTCGTAGATACATTTGCTCC |
| ChipPCR-Lox-1-R | GAGTTGGGTTATCATAACTTCAAC |
| ChipPCR-Lox-2-F | TCTCACAAGCCAAACAAGGA |
| ChipPCR-Lox-2-R | TGAACAACCCTTTCACCCC |
| ChipPCR-UBQ5-F | CCAGCAAAACATAGGCCCAAA |
| ChipPCR-UBQ5-R | TGTGGCTAGCTAGGGTTTCT |
